# Metagenomic binning of a marine sponge microbiome reveals unity in defense but metabolic specialization

**DOI:** 10.1038/ismej.2017.101

**Published:** 2017-07-11

**Authors:** Beate M Slaby, Thomas Hackl, Hannes Horn, Kristina Bayer, Ute Hentschel

**Affiliations:** 1 RD3 Marine Microbiology, GEOMAR Helmholtz Centre for Ocean Research Kiel, Kiel, Germany; 2 Department of Botany II, Julius-von-Sachs Institute for Biological Science, University of Würzburg, Würzburg, Germany; 3 Department of Civil and Environmental Engineering, Massachusetts Institute of Technology, Cambridge, MA, USA; 4 Christian-Albrechts University of Kiel, Kiel, Germany

## Abstract

Marine sponges are ancient metazoans that are populated by distinct and highly diverse microbial communities. In order to obtain deeper insights into the functional gene repertoire of the Mediterranean sponge *Aplysina aerophoba*, we combined Illumina short-read and PacBio long-read sequencing followed by un-targeted metagenomic binning. We identified a total of 37 high-quality bins representing 11 bacterial phyla and two candidate phyla. Statistical comparison of symbiont genomes with selected reference genomes revealed a significant enrichment of genes related to bacterial defense (restriction-modification systems, toxin-antitoxin systems) as well as genes involved in host colonization and extracellular matrix utilization in sponge symbionts. A within-symbionts genome comparison revealed a nutritional specialization of at least two symbiont guilds, where one appears to metabolize carnitine and the other sulfated polysaccharides, both of which are abundant molecules in the sponge extracellular matrix. A third guild of symbionts may be viewed as nutritional generalists that perform largely the same metabolic pathways but lack such extraordinary numbers of the relevant genes. This study characterizes the genomic repertoire of sponge symbionts at an unprecedented resolution and it provides greater insights into the molecular mechanisms underlying microbial-sponge symbiosis.

## Introduction

Marine sponges (Porifera) are evolutionary ancient metazoans dating back to Precambrian times (Li *et al.*, 1998; Love *et al.*, 2009). By filtering extensive volumes of seawater—up to thousands of liters per kg sponge daily (Reiswig, 1974)—they take in food bacteria, but also potential pathogens, toxins and physical stress factors (De Goeij *et al.*, 2009). Rapid cell turnover rates accompanied by extensive detritus production are likely a means of avoiding permanent, stress-induced damage to the sponge (De Goeij *et al.*, 2009; Alexander *et al.*, 2014). In line with the holobiont concept (Bordenstein and Theis, 2015), the highly diverse and distinct symbiotic microbial communities of marine sponges are thought to play a crucial role in their evolutionary success (Easson and Thacker, 2014; Tian *et al.*, 2014; Webster and Thomas, 2016). 16S rRNA gene amplicon studies discovered an unusually high phylum-level diversity and stability of microbial associations in marine sponges comprising phototrophic as well as heterotrophic symbionts (Schmitt *et al.*, 2012a; Easson and Thacker, 2014; Thomas *et al.*, 2016; Webster and Thomas, 2016). The sponge microbiome includes as many as 52 microbial phyla and candidate phyla with the diversity and abundance varying between sponge species (Webster and Thomas, 2016). The most dominant symbiont groups belong to the phyla Proteobacteria (mainly gamma- and alphaproteobacteria), Actinobacteria, Chloroflexi, Nitrospirae, Cyanobacteria, candidatus phylum Poribacteria, and Thaumarchaea (Webster and Thomas, 2016).

Comparisons of sponge-associated and seawater microbial consortia have identified a number of genomic features that seem to facilitate bacterial adaptation to a symbiotic existence within sponges, for example, transposable elements, defense mechanisms, and eukaryote-like proteins (Thomas *et al.*, 2010; Fan *et al.*, 2012; Hentschel *et al.*, 2012; Horn *et al.*, 2016). Studies on individual clades of the microbial consortium have revealed specific features of sponge symbionts, such as adaptations of the lipopolysaccharide by the cyanobacterium ‘*Candidatus* Synechococcus spongiarum’ presumably to avoid host phagocytosis (Gao *et al.*, 2014; Burgsdorf *et al.*, 2015) and specific patterns for carbon degradation by Poribacteria (Kamke *et al.*, 2013).

PacBio long-read sequencing is widely used in isolate genomics as a stand-alone tool or in combination with Illumina short-read sequencing (for example, Beims *et al.*, 2015; Koren and Phillippy, 2015; Ricker *et al.*, 2016). The error-prone PacBio reads need to be corrected either with themselves—if sufficient sequencing depth is provided—or with Illumina reads of far lower error-rate (Koren *et al.*, 2012; Ono *et al.*, 2013). The combination of PacBio and Illumina sequencing data in hybrid assemblies enables the closure of assembly gaps, for example, by spanning over long repeats, to merge contigs and thereby to reconstruct the genome architecture (Koren *et al.*, 2012). In metagenomics, the Illumina-PacBio hybrid assembly approach has recently been shown to improve the quality of assemblies (Frank *et al.*, 2016; Tsai *et al.*, 2016). Although the improvements in targeted binning of the dominant members of the microbiomes in these studies have been demonstrated (Frank *et al.*, 2016; Tsai *et al.*, 2016), un-targeted binning and performance for less abundant members of the microbial communities have not been evaluated.

Even though considerable metagenomic information from sponge microbiomes has been accrued, only few symbiont genomes have been reconstructed by single-cell genomics or binning (Siegl *et al.*, 2011; Kamke *et al.*, 2013; Gao *et al.*, 2014, Burgsdorf *et al.*, 2015). Therefore, correlations between phylogeny and function have only rarely been possible. In the present study, we aimed at obtaining a larger number and greater diversity of sponge symbiont genomes. We present the first metagenomic hybrid assembly derived from Illumina short-read and the PacBio long-read data with subsequent un-targeted differential coverage binning. The highly complex microbiome of the Mediterranean sponge *Aplysina aerophoba* was used towards this goal. By applying an un-targeted binning technique, we aimed to include also the less abundant members of the microbial community. We provide statistical evidence for gene networks that are enriched in the symbiont genomes over selected reference genomes and we discuss the role of these genomic adaptations in context of a symbiotic existence in the sponge matrix. Furthermore, a comparison between symbiont genomes revealed a specialization into three distinct, yet phylogenetically diverse groups within the consortium, of which two appear to metabolize distinct components of the sponge extracellular matrix.

## Materials and methods

### Sample collection

*Aplysina aerophoba* specimens were collected from the Mediterranean Sea near Piran, Slovenia (45.517680, 13.567991). One specimen was collected in May 2013 for Illumina sequencing and one specimen was collected in May 2014 for PacBio sequencing. Both were collected from ca. 5 m depth and transported to the laboratory in natural seawater at ambient temperature. Sponge pinacoderm (outer layer) and mesohyl (inner core), visually distinguishable by the reddish-greenish color of the cyanobacteria-containing pinacoderm, were separated with a sterile scalpel blade and microbial cell enrichment was performed by differential centrifugation (Fieseler *et al.*, 2006). These sponge-associated prokaryotes (SAPs) were frozen with 15% glycerin at −80 °C.

### DNA extraction and sequencing

DNA of sponge-associated prokaryotes (SAPs) obtained from either pinacoderm or mesohyl tissue (three technical replicates each) was extracted with the FastDNA SPIN Kit for Soil (MP Biomedicals, Santa Ana, CA, USA). Different cell lysis protocols were applied for each triplicate to obtain differential sequencing coverage for downstream binning as previously described (Albertsen *et al.*, 2013; Alneberg *et al.*, 2014): (i) bead beating, following the manufacturer’s protocol, (ii) freeze-thaw cycling (3 cycles of 20 min at −80 °C and 20 min at 42 °C), (iii) proteinase K digestion for 1 h at 37 °C (TE buffer with 0.5% SDS and proteinase K at 100 ng ml^−1^ final concentration). Metagenomic DNA was sequenced on an Illumina HiSeq2000 platform (150-bp paired-end reads) and quality filtered at the DOE Joint Genome Institute (Walnut Creek, CA, USA) following the JGI sequencing and the data processing pipeline (Markowitz *et al.*, 2012). Additionally, V4 iTag sequences were obtained by Illumina MiSeq sequencing and analyzed in the respective iTagger pipeline at JGI (for more information, see https://bitbucket.org/berkeleylab/jgi_itagger and http://jgi.doe.gov/wp-content/uploads/2013/05/iTagger-methods.pdf). For the PacBio data set, DNA was extracted with the above-mentioned kit following the manufacturer’s protocol (cell lysis by bead beating) and sequenced on a PacBio RS II platform using 8 SMRT cells by GATC Biotech (Konstanz, Germany).

### Assembly, binning, and annotation

Illumina reads were coverage-normalized with bbnorm of BBMap v. 34 (https://sourceforge.net/projects/bbmap/) at default settings. PacBio reads were corrected with all (non-normalized) Illumina reads using proovread (Hackl *et al.*, 2014) optimized for handling the metagenomic data (Hackl, 2016). Only corrected PacBio reads longer than 1000 bp were used for further analyses. To assess the improvement of the assembly by adding PacBio long-reads compared to only Illumina short-reads, we assembled two sets of data as follows: (i) only the Illumina reads (Illumina-only assembly) and (ii) Illumina and PacBio reads together (hybrid assembly). The two independent assemblies were calculated with SPAdes v. 3.5.0 (Bankevich *et al.*, 2012) for kmers 21, 33, 55, 77, 99 and 127, and with the single-cell and only-assembler options enabled. Illumina-only contigs and corrected PacBio reads were both mapped to the hybrid assembly with blasr v. 1.3.1 (Chaisson and Tesler, 2012) to assess whether all available information was incorporated into the hybrid assembly. Only contigs of at least 1000 bp length were used for further analyses.

Binning was performed with CONCOCT v. 0.4.0 (Alneberg *et al.*, 2014). For this, the data were prepared as follows. Contigs longer than 20 000 bp were split into sub-contigs of at least 10 000 bp length with the provided script (Alneberg *et al.*, 2014). The non-normalized Illumina reads of the six Illumina data sets were mapped to the sub-contigs with bowtie2 v. 2.2.2 at default settings (Langmead and Salzberg, 2012). The resulting SAM files were converted to BAM, sorted and indexed with samtools v. 0.1.18 (Li *et al.*, 2009), and duplicates were marked according to the script map-bowtie2-markduplicates.sh provided with the CONCOCT package (Alneberg *et al.*, 2014). Samtools v. 0.1.18 was also used for depth calculation (Li *et al.*, 2009). The in-house python script avgcov_from_samtoolsout.py (https://github.com/bslaby/scripts/) was used to calculate the average coverage of each sub-contig. The coverage tables for each mapping were merged into one for binning with CONCOCT v. 0.4.0 (Alneberg *et al.*, 2014) at default settings. A fasta file for each bin was created with the in-house python script mkBinFasta.py (https://github.com/bslaby/scripts/). Sub-contigs were merged into the original contigs again. If sub-contigs of one contig were assigned to different bins, the contig was placed in the bin by majority-vote. Assembly statistics were obtained from QUAST v. 3.1 (Gurevich *et al.*, 2013). To assess similarity of Illumina-only and hybrid assembly as well as assembly improvements by adding of PacBio long-reads on the genome level, the contigs of an Illumina-only bin were mapped to the contigs of the corresponding hybrid assembly bin with nucmer of MUMmer 3.0 (Kurtz *et al.*, 2004) and visualized with AliTV (Ankenbrand *et al.*, 2017).

Open reading frames (ORFs) were called with prodigal v. 2.6.1 (Hyatt *et al.*, 2010) with -m and -p meta options enabled, and the completeness of genomic bins was estimated by hmmsearch (HMMER 3.1b1) against a database of 111 essential genes with –cut_tc and –notextw options (Finn *et al.*, 2011; Albertsen *et al.*, 2013). Contamination levels were assessed by a blastp search (BLAST 2.2.28+) of the essential genes against the refseq_protein database (release number 81) at an e-value cutoff of 1e-5 followed by determination of the last common ancestor for each gene by MEGAN version 6.4.3 (Pruitt *et al.*, 2007; Camacho *et al.*, 2009; Huson *et al.*, 2016). Only reference genomes>90% and bins>70% completeness were used in further analyses.

The Illumina-only and the PacBio-Illumina hybrid assemblies were deposited on MG-RAST (Meyer *et al.*, 2008) (Table 1). Additionally, the raw Illumina sequencing data were deposited under GOLD Study ID Gs0099546 (Reddy *et al.*, 2014). Uncorrected and corrected PacBio reads were deposited on MG-RAST (Meyer *et al.*, 2008) with the IDs mgm4670967.3 and mgm4670966.3, respectively. The accession numbers for all bins >70% completeness are listed in Table 2. The Illumina-only assembly is also deposited on GenBank with the accession MKWU00000000.

### Comparative analysis

A total of 27 reference genomes were chosen based on phylogeny and environment (Supplementary Table 1). Close taxonomic relatedness to the symbiont genomes, closed genomes, as well as marine (or at least aquatic) environments were preferably selected. In order to be able to validate the binning process, we included the sponge symbiont genomes ‘*Ca*. S. spongiarum’ 15L (Burgsdorf *et al.*, 2015) and ‘*Ca*. Poribacterium’ (Kamke *et al.*, 2013) in the analyses. We retrieved nucleic acid fasta files for all selected references from GenBank and MG-RAST (Benson *et al.*, 2007; Meyer *et al.*, 2008), which were then processed like the symbiont bins with respect to ORF prediction and annotation. Five additional references were added for 16S rRNA gene tree calculation for better phylogenetic resolution (see Supplementary Figure 3A). The annotation of rRNA genes was performed with rRNA prediction at default settings (Wu *et al.*, 2011). The 16S rRNA genes were taxonomically assigned using the RDPclassifier at a 80% confidence cutoff (Wang *et al.*, 2007) and the classification tool of SINA 1.2.11 (Pruesse *et al.*, 2012) using the SILVA and Greengenes databases (DeSantis *et al.*, 2006; Quast *et al.*, 2013). Gap-only sites were removed from the SINA alignment of both, bins and references, in SeaView 4.5.2 (Gouy *et al.*, 2010). A Neighbor Joining tree (GTR+G+I), which was determined to be the most suitable DNA/protein model for the data, was calculated in MEGA7 with 100 bootstrap replications (Kumar *et al.*, 2016). Additionally, a concatenated gene tree of 29 essential genes was created (see Supplementary Table 2 for a list of genes). Alignments for every gene individually using the muscle algorithm in MEGA7 (Edgar, 2004; Kumar *et al.*, 2016) were merged with a sequence of 20 Ns between the genes. After identifying the most suitable DNA/protein model for the data, a maximum likelihood tree (LG+G+I) was calculated in MEGA7 with 100 bootstrap replications (Kumar *et al.*, 2016). Bins lacking 16S rRNA genes or with an ambiguous classification of this gene were phylogenetically classified according to their placement in the concatenated tree.

To assess the distribution of the binned sponge symbionts among different sponge species, a BLAST search was conducted for the available 16S rRNA genes against a database of the representative amplicon OTUs by Thomas *et al.* (2016). For each bin, the best three hits were obtained, the sequences were aligned with SINA, and a Neighbor Joining tree (K2P) was calculated in SeaView with 1000 bootstrap replications (Gouy *et al.*, 2010; Pruesse *et al.*, 2012). The closest OTU for each bin was determined based on BLAST results and the phylogenetic tree. Information on the distribution of the selected OTUs was obtained from Thomas *et al.* (2016).

ORFs were annotated with rpsblast+ of BLAST 2.2.28+ against a local version of the COG database (ftp://ftp.ncbi.nih.gov/pub/mmdb/cdd/, download on 28 May 2015) (Tatusov *et al.*, 2003; Camacho *et al.*, 2009). Only annotations with an e-value⩽1e-6 were used for further analyses, and only one annotation per ORF was kept ranked by e-value, length and bitscore. Because many sponge–symbiont lineages, in some cases whole phyla, are not abundant in seawater, we have opted for an approach different from previous publications, where only seawater metagenomes were used for comparison (for example, Thomas *et al.*, 2010). We selected reference genomes based on phylogenetic similarity and on genome completeness. Marine sources were preferred over other sources.

To discover statistically significant differences between the sponge symbiont genomes and reference genomes, Welch’s *t*-test was performed in STAMP 2.0.9 (Parks *et al.*, 2014) with Storey FDR and a q-value cutoff of 0.01. This was performed on the COG class level, double-counting COGs that belong to multiple classes, as well as on the COG level. Interactions between the significantly sponge-enriched COGs were explored using STRING v10 networks (Szklarczyk *et al.*, 2014) and a heatmap was created in R version 3.2.3 (https://www.r-project.org). The phylo.heatmap function of phytools package version 0.5.30 (Revell, 2012) was used to complement the heatmap with phylogeny. The phylogenetic tree accompanying the heatmap is a simplified version (bins only) of the concatenated gene phylogeny.

The symbiont genomes were compared by applying a principle component analysis (PCA) in R with FactoMineR package version 1.33 (Lê *et al.*, 2008), factoextra package version 1.0.3 (https://cran.r-project.org/web/packages/factoextra/index.html), and ggplot2 version 2.2.0 (http://ggplot2.org).

## Results and discussion

Two metagenome assemblies were obtained, one only from Illumina HiSeq short-reads (Illumina-only assembly), and one from the same Illumina short-reads set, but combined with pre-corrected PacBio long-reads (hybrid assembly). The two assemblies differed notably in number of contigs and total size (Table 1). The Illumina-only assembly comprised >100 000 contigs with a total length of 490 Mbp, the hybrid assembly consisted of >30 000 contigs with a total length of 301 Mbp. Mappings of all contigs of the Illumina-only assembly and the corrected PacBio reads to the hybrid assembly showed that 100% of each data set mapped to the hybrid assembly. This demonstrates that all information had been transferred to the hybrid assembly. The hybrid assembly is smaller because it is better resolved, thus leading to a lower number of ambiguities. Only contigs >=1000 bp were considered. The addition of the PacBio reads to the assembly increased the N_50_ value 3.8-fold, from about 9 kbp to 34 kbp. While the number of highly complete genome bins (>70% completeness) decreased (42 Illumina-only bins vs 37 hybrid bins), the portion of full-length 16S rRNA gene containing bins doubled from 16 in the Illumina-only assembly to 32 in the hybrid assembly. To assess if contigs from the Illumina-only assembly were reappearing in the hybrid assembly and if the PacBio reads merged them into larger contigs, an Illumina-only bin was mapped to the corresponding hybrid bin. This allowed a visual comparison of the assemblies (Supplementary Figure 1). This mapping shows that the two assemblies corresponded well because contigs that had been constructed out of the Illumina data reappeared upon addition of the PacBio reads. Moreover, they were merged into even larger contigs, thus resulting in a higher-quality bin.

To obtain the short-read data optimized for differential coverage binning, six DNA samples from the same sponge specimen were extracted with varied lysis protocols, and deeply sequenced on an Illumina HiSeq2000 instrument (see Supplementary Figure 2 of JGI Project ID 1024999 for the additional ribosomal 16S rRNA V4 iTag data of this sequencing project). Although we already obtained a large number of high-completeness bins from the Illumina-only assembly, only 38% of the binned genomes contained a 16S rRNA gene. Contrasting, in the PacBio-Illumina hybrid assembly 86% of the bins contained a 16S rRNA gene (Table 1). Furthermore, with a 3.8-fold higher N_50_ hybrid assembly was more contiguous. For these reasons, all downstream analyses were carried out with the genomes binned from the PacBio-Illumina hybrid assembly.

### Thirty-seven high-quality sponge symbiont genomes representing 13 bacterial phyla

The 37 binned genomes belonged to 11 bacterial phyla and 2 candidate phyla, which are representative of the sponge symbiont consortium: Proteobacteria (Alpha, Gamma, and Delta), Chloroflexi, Acidobacteria, Actinobacteria, Bacteroidetes, Gemmatimonadetes, Deinococcus-Thermus, Nitrospirae, Nitrospinae, Cyanobacteria, Spirochaetes and the candidate phyla Poribacteria and SBR1093 (Table 2). This composition is congruent with the known microbial diversity of *A. aerophoba* (Hentschel *et al.*, 2002; Schmitt *et al.*, 2012b). The bins varied in total number of contigs from 21 to 758. Large numbers of contigs did not correlate with low sequence coverage: the bin with lowest coverage (bin18 with 38-times coverage), for example, was composed of as few as 83 contigs and was 87% complete. Estimated genome sizes, based on total length and estimated genome completeness, ranged from 1.9 Mbp (Alphaproteobacterium bin98) to 7.9 Mbp (Acidobacterium bin110). With respect to GC content, the genomes ranged from 36% (Bacteroidetes bin25) to nearly 70% (Alphaproteobacterium bin129). Overall, the sponge symbionts had genomes of high GC-content, which are as follows: 13 were between 50 and 60%, 17 of symbiont genomes comprised >60% of GC-bases. Comparably high average GC contents are a known feature of sponge metagenomes (Horn *et al.*, 2016). The N_50_ values also showed variability, with the smallest being 6974 bp for Alphaproteobacterium bin95 and the largest being 309 970 bp for Chloroflexi bin127. The number of coding sequences (CDSs) in the symbiont genomes ranged from 1455 (Alphaproteobacterium bin98) to 6288 (Ca. Poribacterium bin44). The number of COGs annotated for each genome ranged between 490 (bin98) and 3450 (Alphaproteobacterium bin129), which translates to 34% (bin98) and 76% (Alphaproteobacterium bin65) CDSs in COGs.

**Figure 1:**
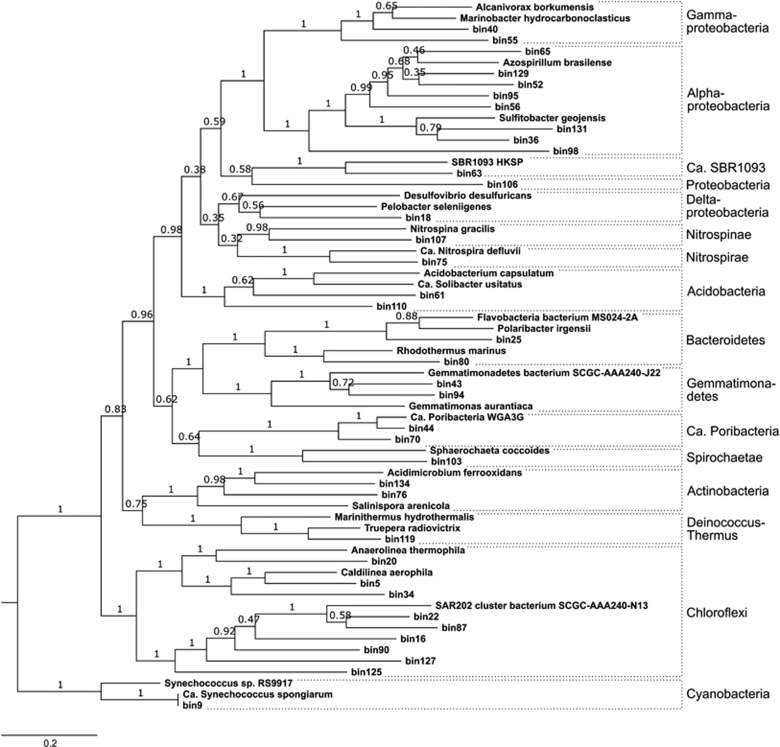
Maximum likelihood (LG+G+I) phylogenetic tree based on the amino acid sequences of 29 essential genes, calculated in MEGA7 with 100 bootstrap replications. Cyanobacteria were used as outgroup, because they were closest to the archaeal outgroup in the 16S rRNA gene phylogeny (Supplementary Figure 3A).

In order to resolve the phylogenies of the recovered bins, a concatenated tree (Figure 1) of 29 essential single-copy genes (Supplementary Table 2) as well as a 16S rRNA gene tree were constructed (Supplementary Figure 3A). Overall, the phylogeny of the binned bacterial genomes reflected the major phylogenetic lineages known to inhabit sponges (Supplementary Figure 3B; Thomas *et al.*, 2016). This finding suggests that the sequenced lineages are prevalent in *A. aerophoba*, as more abundant taxa were more likely sequenced than rare lineages from this diverse metagenome. Our hypothesis that the binned genomes derive from symbionts and not from environmental bacteria was further supported by the 16S rRNA gene data. The best BLAST hits for all 34 bin-derived 16S rRNA genes were from sponge-associated or sponge/coral-associated bacteria (Supplementary Table 3A). Because the remaining three bins did not contain a 16S rRNA gene, their identity could not be confirmed by BLAST alone.

The concatenated tree shows the phylogenetic placement of all 37 bins and their references, which had been selected based on genome completeness, phylogenetic similarity, and habitat (marine preferred over other habitats) (Supplementary Tables 1 and 3B). It was in overall agreement with the 16S rRNA gene tree regarding the phylogenetic placement of the bins containing this gene and furthermore provides placement for the three bins missing the 16S rRNA gene.

### Sponge symbiont genomes are enriched in defense and in matrix interactions

In order to identify the gene functions that are enriched in the genomes of sponge symbionts, we compared the pool of symbiont genomes against the pool of selected reference genomes. Significant differences were identified between the symbiont genomes and reference genomes on the level of COG classes. While COG classes R (‘General function prediction only’), E (‘amino acid transport and metabolism’), L (‘replication, recombination and repair’), and Q (‘secondary metabolites biosynthesis, transport and catabolism’) are enriched in the symbionts, the classes T (‘signal transduction mechanisms’), K (‘transcription’), M (‘cell wall/membrane/envelope biogenesis’) and N (‘cell motility’) were depleted in comparison to the reference genomes (Supplementary Figure 4A).

**Figure 2:**
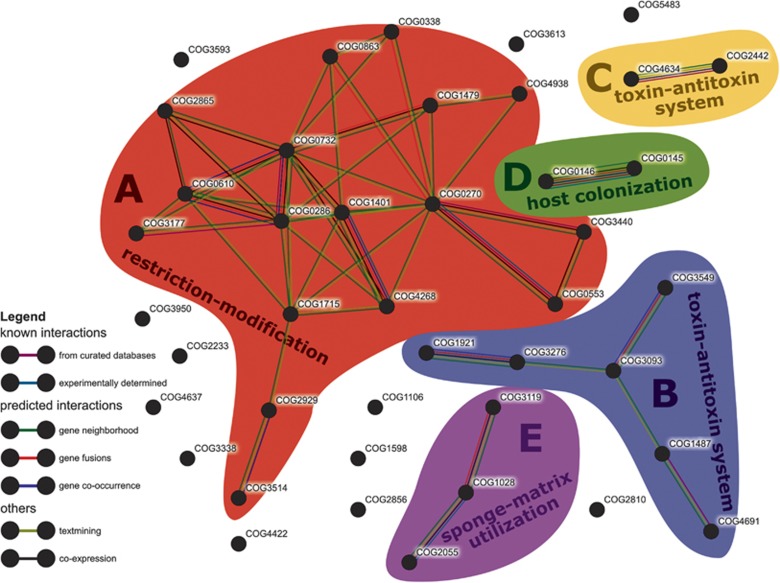
STRING network of significantly sponge symbiont-enriched COGs. Colored areas mark COGs that belong to the same network (A–E). Colors of the connectors indicate the type of evidence of the predicted interaction between the two connected COGs. Only connections of ‘high confidence’ (minimum required interaction score: 0.700) are shown.

When comparing on the level of individual COGs, 42 symbiont-enriched genes were identified (Supplementary Figure 4B). Most of them (43%) belonged to COG classes R and S (‘general function prediction only’ and ‘function unknown’), a large fraction (19%) belonged to class V (‘defense mechanisms’), and 5 (12%) to class L (‘replication, recombination and repair’). According to the STRING database, many of these significantly symbiont-enriched COGs were likely interacting (Figure 2). At a high confidence cutoff (0.700 minimum required interaction score), five networks (A–E) comprising 17, 6, 3, 2 and 2 COGs were obtained. The remaining 12 symbiont-enriched COGs did not interact with any other COGs in the list. The set includes a restriction endonuclease (COG2810) and a bacteriophage protein gp37 (COG4422).

**Figure 3:**
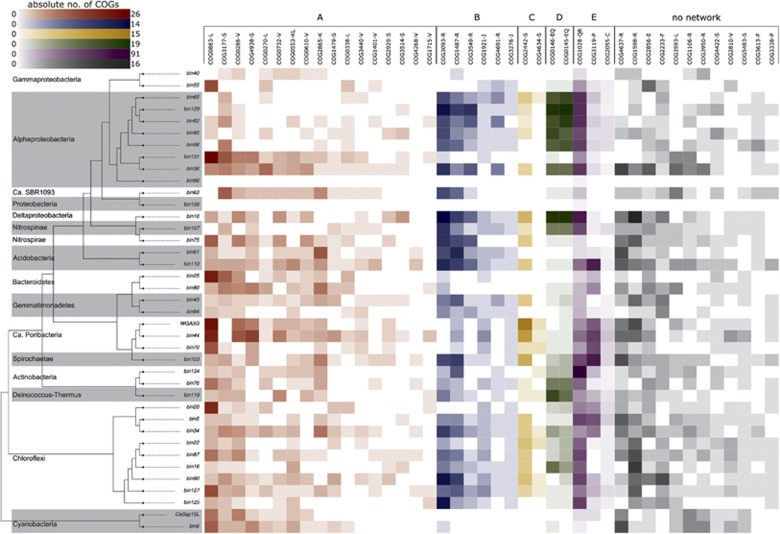
Heatmap of absolute counts of significantly sponge symbiont-enriched COGs in the genomes binned from the PacBio-Illumina hybrid assembly. Phylogenetic relationships of the genomes are indicated by a simplified version of the tree in Figure 1 (only sponge symbionts are shown here). Possibly interacting COGs as shown in Figure 2 are grouped and colored accordingly and marked by the letters A–E. The letters next to each COG indicate the according COG class.

The largest STRING network was built of sponge-enriched COGs related to restriction-modification (RM) with endonucleases, helicases and methylases (cluster A in Figure 2, see Supplementary Table 4 for COG annotation). It was present in all sponge symbiont phyla in this study (Figure 3). RM systems represent one major line of defense against incoming, foreign DNA, a feature frequently referred to as bacterial immunity (Vasu and Nagaraja, 2013). RM systems are also known to play a role in symbioses (for example, Zheng *et al.*, 2016) and have recently also been described in sponge symbionts (Tian *et al.*, 2015; Gauthier *et al.*, 2016; Horn *et al.*, 2016). Many of the COGs of network A were previously described as sponge-enriched (Thomas *et al.*, 2010; Fan *et al.*, 2012; Gao *et al.*, 2014; Burgsdorf *et al.*, 2015). This recurring finding of RM in symbionts of a variety of sponges from different geographic locations, and the abundance of RM in all 13 bacterial phyla in our data set underscore the apparent significance for sponge symbioses.

Most COGs of STRING network B were related to toxin–antitoxin (TA) systems that supposedly play a role in phage defense, stress response, and programmed cell death (for example, Sberro *et al.*, 2013). COG3549 and COG3093 form the HigAB TA plasmid maintenance system, and COG1487 encodes for the toxin in a TA system of the VapBC family (Makarova *et al.*, 2009; Sberro *et al.*, 2013). COG4691 is a plasmid stability protein and encodes for a proposed antitoxin of a VapBC TA system (Chen, 2007). COG1921 (SelA) and COG3276 (SelB), involved in selenocysteine production (Stolz *et al.*, 2006), co-occurred in the majority of symbiont bins of various phyla but were missing in the majority of their closely related references (Supplementary Table 4). STRING network C consists of COG4634 and COG2442, two uncharacterized conserved proteins according to the NCBI annotation. COG4634 is hypothesized to be a fine-tuning modulator in conjugative plasmid transfer (López-Fuentes *et al.*, 2015), and COG2442 is a PIN-associated antitoxin in a widespread TA system most abundant in Cyanobacteria and Chloroflexi (Makarova *et al.*, 2009). Furthermore, COG2929 and COG3514, which are part of network A, were predicted to form a TA system as well (Makarova *et al.*, 2009). Both COGs co-localize on a plasmid of the cyanobacterium *Synechococcus elongatus* PCC7942 where this TA system plays a crucial role in plasmid maintenance (Chen, 2007). In our data set, both COGs co-occurred in 16 sponge symbiont bins of various bacterial phyla, but only once in the reference group, in the acidobacterium *Solibacter usitatus*. The abundance and distribution of multiple RM and TA systems in their genomes suggests that defense against foreign DNA is an important feature of sponge symbionts being consistent with the previously stated concept of their convergent evolution (Thomas *et al.*, 2010; Fan *et al.*, 2012; Horn *et al.*, 2016). These defense mechanisms are possibly a necessary countermeasure against the exposure to free DNA resulting from the sponge’s extensive filtration and phagocytosis activity (Reiswig, 1974).

Symbiont-enriched STRING networks D and E are related to colonization of the host and possibly utilization of the host matrix. COG0145 (hyuA) and COG0146 (hyuB) of network D have been hypothesized to play an important role for *Helicobacter pylori* in the colonization of mice (Zhang *et al.*, 2009). These genes are known to be involved in the metabolism of hydantoin (Kim *et al.*, 2000). The abundance and distribution of network D across various phyla of sponge-associated bacteria in our study suggests that it may also be of importance for the colonization of sponge hosts. COG1028 (FabG) and COG3119 (arylsulfatase A) of network E displayed the highest counts within the sponge-enriched COGs. Arylsulfatase A might allow the symbionts to metabolize sulfated polysaccharides from the sponge extracellular matrix, where their abundance has been documented in a number of sponge species, including the related species *Aplysina fulva* (Zierer and Mourão, 2000; Vilanova *et al.*, 2009).

### Sponge symbionts display metabolic specialization

**Figure 4:**
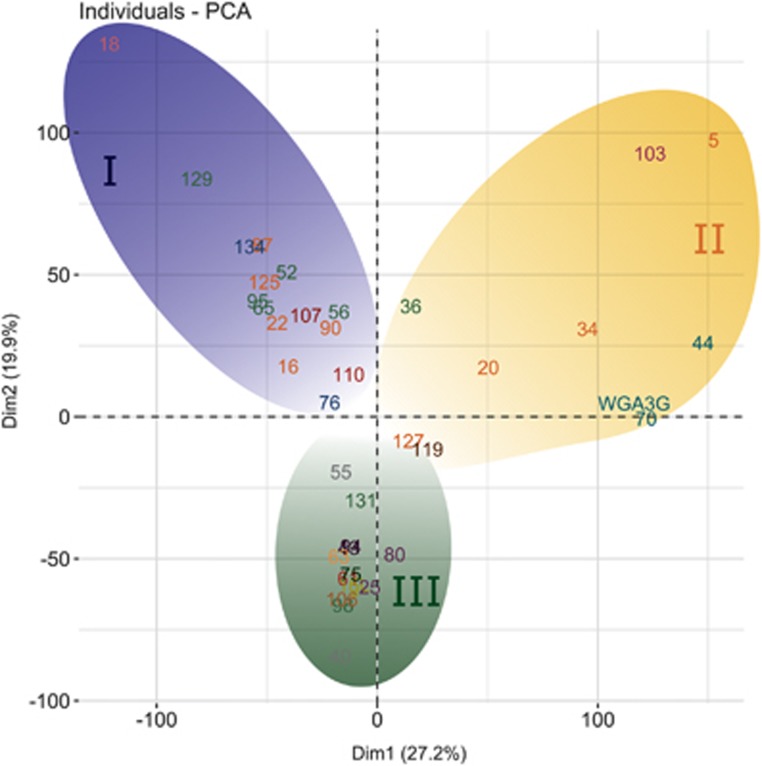
PCA plot comparing the genomes of the sponge-symbionts to each other based on their COG annotation. Phylogenetic affiliation is indicated by font colors (see Table 2 for details). The symbionts build three groups I–III marked by background color (blue, yellow and green, respectively).

**Figure 5:**
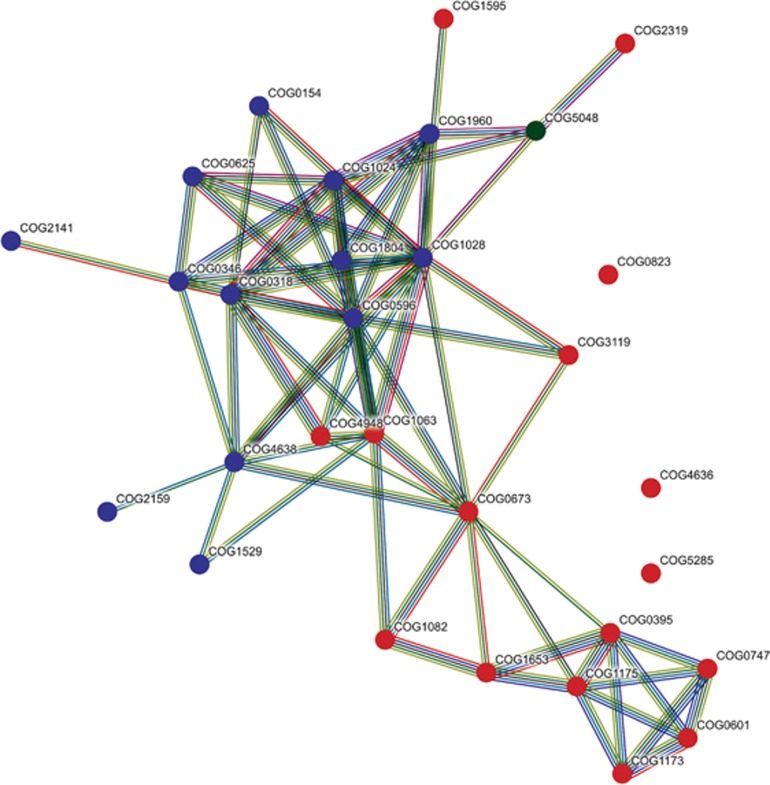
STRING network of the 30 COGs contributing most to the grouping of the sponge-symbionts in Figure 4. Circles representing the COGs’ position in the network are colored according to the symbiont group where they are overrepresented (group I–III: blue, red and green, respectively). Colors of the connectors indicate the type of evicence of the predicted interactions between the two connected COGs as shown in Figure 2. Only connections of ‘high confidence’ (minimum required interaction score: 0.700) are shown.

**Figure 6:**
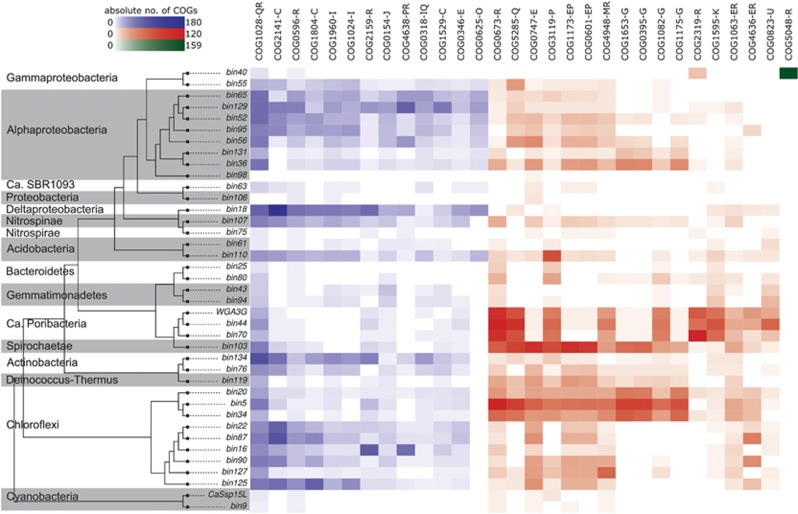
Heatmap of absolute counts of the 30 COGs contributing most to the grouping of the sponge-symbionts as shown in Figure 4. Phylogenetic relationships of the genomes are indicated by a simplified version of the tree in Figure 1 (only sponge symbionts are shown here). Colors represent the symbiont group, where the regarding COGs is overrepresented (group I–III: blue, red and green, respectively). The letters next to each COG indicate the according COG class.

In order to compare the symbiont genomes among each other and to identify functional groups, a principle component analysis (PCA) was performed, clustering the sponge symbiont clades into three functional groups (Figure 4). The 30 COGs with the greatest influence on the functional grouping are shown in Supplementary Figure 5. Most COGs of symbiont groups I, II and III are strongly connected according to a STRING network with the COGs enriched in groups I and II clustering on different sides of the network (Figure 5). The correlation between the 30 COGs and their phylogenetic context is shown in Figure 6. The functional grouping is only partly coherent with phylogeny. While for example, Gemmatimonadetes cluster closely together, Chloroflexi are split up in two groups, which are as follows: (i) SAR202 clustering with a group of Alphaproteobacteria, Deltaproteobacteria, Nitrospinae and Actinobacteria, and (ii) Caldilineae and Anaerolineae that built a group with Poribacteria and Spirochaetae.

According to our analysis, the COGs enriched in symbiont group I are mainly involved in metabolism and energy production. Most enriched in this group are COGs related to carnitine metabolism. Carnitine is an organic compatible solute that some bacteria can use as a source for carbon, nitrogen, and energy (Meadows and Wargo, 2015). It is produced by most eukaryotes, including sponges (Fraenkel, 1954) and we posit that it may be taken up by symbiotic bacteria from the readily available sponge-derived detritus consisting largely of shed sponge cells (De Goeij *et al.*, 2009; Alexander *et al.*, 2014). Uptake of carnitine by bacteria can also serve as protection against environmental stress like variation in water content, salinity, or temperature (Meadows and Wargo, 2015).

Symbiont group II is characterized by high numbers of arylsulfatase A genes (COG3119), various ABC transporters and dehydrogenases. This phylogenetically heterogeneous guild of microorganisms seems to be specialized on the utilization of sulfated polysaccharides, as described above for symbiont-enriched COG network E. Inspection of the genomic context on the bin-level shows that the arylsulfatase repeatedly clusters with the ABC transporters and the dehydrogenase that are likewise enriched in symbiont group II (Supplementary Figure 6 and Supplementary Table 5). This further supports our hypothesis that this gene cluster is of importance for sponge symbionts, and especially for the members of symbiont group II.

The genomes of symbiont group III did not show an enrichment of any particular COGs. They also contained the COGs of symbiont groups I and II, but not in as high numbers. We therefore posit that symbiont group III is not metabolically specialized and may represent a group of metabolic generalists. Within the 30 COGs most responsible for the grouping, only COG5048 (FOG: Zinc-finger) was enriched in bin40 of this group with a total of 159 copies. Zn-fingers are small structural protein motifs that have general cellular roles in binding nucleic acids and proteins. They are commonly found in eukaryotes, but also present in prokaryotes, where they are likely involved in virulence or symbiosis (Malgieri *et al.*, 2015).

## Conclusions

The complementation of Illumina short-read with PacBio long-read sequencing for metagenomic binning of highly complex environmental samples greatly improves the overall assembly statistics. It also improves the quality of binned genomes and eases, often newly enables phylogenetic classification of the binned genomes. The statistical comparison revealed an enrichment of genes related to RM and TA systems in most symbiont genomes over the reference genomes. This implies that the defense against incoming foreign DNA is of high importance for a symbiotic existence within the sponge mesohyl. This finding is particularly relevant in the context of the extensive animal’s filtration and phagocytosis activities, resulting in an ample exposure of the symbionts to free DNA. Secondly, host colonization and host matrix utilization were identified as significantly enriched features in sponge symbionts. The within-symbionts genome comparison revealed a nutritional specialization, where one guild of symbionts appears to metabolize carnitine, while the other appears to metabolize sulfated polysaccharides, both of which are abundant molecules of the sponge extracellular matrix. We hypothesize that the sponge symbionts feed on the sponge cells that are shed as part of the cell turnover, and on components of the sponge extracellular matrix. A third guild of symbionts may be viewed as nutritional generalists, whose precise function within this consortium remains to be identified. The presence of guilds specializing on different metabolic traits, such as butyrate or lactate production or mucin degradation, is also known from the human gut microbiome (Shetty *et al.*, 2017). Complex microbial communities may structure themselves around nutritional niches provided by the specific host-related environment. The unprecedented resolution of the genomic repertoire was enabled by binning of a metagenomic hybrid assembly of hitherto unprecedented depth for sponge symbioses. The hypotheses on niche specializations by the symbionts could be tested in feeding studies combined with metatranscriptomics and/or imaging techniques.

## Figures and Tables

**Table 1:** Comparison of Illumina-only and Illumina-PacBio hybrid assemblies.

	*Illumina-only*	*Illumina-PacBio hybrid*
MG-RAST ID	mgm4671062.3	mgm4671058.3
Contig number (⩾1000 bp)	110 609	31 187
Size (Mb)	490	301
N50	8958	33 831
N75	2873	12 184
L50	8886	1980
L75	34 979	5726
CDSs	509 054	289 685
Bin number	217	137
>90% completeness (with 16S rRNA gene)	25 (12)	26 (22)
85–90% completeness (with 16S rRNA gene)	12 (4)	6 (6)
70–85% completeness (with 16S rRNA gene)	5 (0)	5 (4)

**Table 2:** Binned genomes of Illumina-PacBio hybrid assembly

*Accession*	*Bin*	*Phylogeny*	*Symbiont guild*	*% est. com.*	*Contig no.*	*Times cov.*	*Est. size (Mb)*	*% GC*	*N50*	*CDS no.*	*% in COGs*	*Dupl.*
MPNP00000000	bin131	Proteobacteria; Alphaproteobacteria; Rhodobacterales; Rhodobacteraceae^a^	III	93.69	416	612	4	41.99	19 278	3392	59.58	1
MPMP00000000	bin36	Proteobacteria; Alphaproteobacteria; Rhodobacterales; Rhodobacteraceae; Albidovulum^a^	II	89.19	201	189	6.3	58.04	44 410	5122	64.02	0
MPMX00000000	bin65	Proteobacteria; Alphaproteobacteria; Rhodospirillales; Rhodospirillaceae; uncultured^b^	I	91.89	94	396	4.7	66.16	72 338	4036	76.39	0
MPNO00000000	bin129	Proteobacteria; Alphaproteobacteria; Rhodospirillales; Rhodospirillaceae; Defluviicoccus^b^	I	82.88	122	237	5.8	69.54	56 772	4742	72.75	0
MPMV00000000	bin56	Proteobacteria; Alphaproteobacteria; Rickettsiales; EF100-94H03^b^	I	93.69	102	54	4.8	63.69	78 682	4292	70.36	0
MPMT00000000	bin52	Proteobacteria; Alphaproteobacteria^c^	I	92.79	120	234	4.5	66.54	52 938	3989	72.75	1
MPNG00000000	bin98	Proteobacteria; Alphaproteobacteria^a^	III	85.59	105	96	1.9	40.65	46 493	1455	33.68	0
MPNF00000000	bin95	Proteobacteria; Alphaproteobacteria^c^	I	75.68	582	152	4.5	66.27	6974	3890	64.16	0
MPMI00000000	bin18	Proteobacteria; Deltaproteobacteria; Desulfurellales; Desulfurellaceae; uncultured^b^	I	87.39	83	38	6	57.83	103 191	5238	65.65	0
MPMQ00000000	bin40	Proteobacteria;Gammaproteobacteria;Oceanospirillales;Hahellaceae;Kistimonas;^a^	III	93.69	215	76	4	57.27	38 525	2848	46.91	0
MPMU00000000	bin55	Proteobacteria; Gammaproteobacteria^a^	III	84.68	183	47	3.5	47.27	24 711	2562	67.49	1
MPNI00000000	bin106	Proteobacteria^a^	III	90.09	148	53	2.9	39.61	55 882	2088	41.62	0
MPNJ00000000	bin107	Nitrospinae/tectomicrobia group; Nitrospinae^c^	I	90.09	60	440	4.9	59.46	165 774	4046	69.7	1
MPMZ00000000	bin75	Nitrospirae; Nitrospira; Nitrospirales; Nitrospiraceae; Nitrospira;^b^	III	91.89	115	65	3.3	56.24	44 884	3093	56.58	2
MPMH00000000	bin63	SBR1093;EC214;^d^	III	88.29	150	479	2.6	50.41	30 980	2180	68.21	4
MPNK00000000	bin110	Acidobacteria; Holophagae; Subgroup 10; TK85^b^	I	79.28	758	549	7.9	67.45	12 332	5726	55.43	4
MPMW00000000	bin61	Acidobacteria; Acidobacteria^a^	III	70.27	207	117	4.1	67.65	19 828	2561	55.92	0
MPMY00000000	bin70	Candidatus Poribacteria;^c^	II	91.89	106	351	5.5	40.34	70 347	4254	59.07	6
MPMS00000000	bin44	Candidatus Poribacteria; Poribacteria genera incertae sedis^a^	II	91.89	465	265	7.7	47.18	23 989	6288	54.28	10
MPNB00000000	bin80	Bacteroidetes; Cytophagia; Order II; Rhodothermaceae; uncultured^b^	III	92.79	192	453	4.4	50.96	34 696	3555	50.44	0
MPMN00000000	bin25	Bacteroidetes; Flavobacteriia; Flavobacteriales; Flavobacteriaceae^a^	III	90.09	124	589	3.3	36.18	40 599	2420	51.03	0
MPMR00000000	bin43	Gemmatimonadetes; Gemmatimonadetes; BD2-11 terrestrial group^b^	III	92.79	65	633	4.8	67.96	132 700	3664	60.84	1
MPNE00000000	bin94	Gemmatimonadetes^c^	III	91.89	83	190	4.8	66.9	89 414	3702	59.62	1
MPNH00000000	bin103	Spirochaetae; Spirochaetes; Spirochaetales; Spirochaetaceae^a^	II	87.39	96	66	5.5	67.36	71 825	4338	67.75	0
MPNA00000000	bin76	Actinobacteria; Acidimicrobiia; Acidimicrobiales; OM1 clade^b^	I	90.09	82	102	3.9	61.59	108 948	3269	65.65	0
MPNQ00000000	bin134	Actinobacteria; Acidimicrobiia; Acidimicrobiales; Sva0996 marine group^b^	I	90.09	77	224	4.1	64.29	91 761	3487	68.71	1
MPNL00000000	bin119	Deinococcus-Thermus; Deinococci;Deinococcales; Trueperaceae; Truepera^a^	II/III	91.89	91	62	3.5	62.23	62 429	2876	69.47	1
MPMK00000000	bin9	Cyanobacteria; ;SubsectionI; FamilyI; uncultured^b^	III	89.19	391	157	3	58.71	12 771	2808	50.68	2
MPML00000000	bin5	Chloroflexi; Caldilineae; Caldilineales; Caldilineaceae; uncultured^b^	II	92.79	120	81	6	58.5	64 429	4593	68.26	1
MPMO00000000	bin34	Chloroflexi; Caldilineae; Caldilineales; Caldilineaceae; uncultured^b^	II	90.99	111	46	5.1	63.15	63 615	3982	63.01	0
MPMM00000000	bin22	Chloroflexi; SAR202 clade^c^	I	90.99	58	94	4.8	59.2	163 655	4049	57.08	4
MPNN00000000	bin127	Chloroflexi; SAR202 clade^c^	II/III	90.09	21	74	3.3	56.35	309 970	2976	59.98	0
MPND00000000	bin90	Chloroflexi; SAR202 clade^b^	I	89.19	213	61	5.2	57.14	50 603	4453	54.91	5
MPMJ00000000	bin16	Chloroflexi; SAR202 clade^b^	I	88.29	101	70	3.7	65.63	62 928	3253	60.62	0
MPNC00000000	bin87	Chloroflexi; SAR202 clade^c^	I	90.99	67	331	5.4	62.79	269 076	4711	55.47	1
MPNM00000000	bin125	Chloroflexi^a^	I	92.79	66	757	4	62.27	125 355	3410	61.73	1
MPMG00000000	bin20	Chloroflexi^a^	II	91.89	22	245	4	59.31	250 998	3218	72.5	2

Abbreviations: dupl, duplicates; com, completeness; cov, coverage; est, estimated; Phylogenetic information.

Only duplicate genes other than PF00750, PF01795, and TIGR00436 were counted, as these genes are known to occur in multiple copies (Albertsen *et al.*, 2013).

a RDPclassifier; d concatenated gene tree.

b LCA SILVA (SINA).

c concatenated gene tree + 16S rRNA gene tree.

d LCA greengenes (SINA).
