# Supplementary material for: Metagenomic binning of a marine sponge microbiome reveals unity in defense but metabolic specialization
Source: ISME J. 2017 Jul 11;11(11):2465–78. doi: 10.1038/ismej.2017.101 (PMC5649159; doi:10.1038/ismej.2017.101)
Supplement: Supplementary Figure S1 [file ismej2017101x1.docx]

**Figure S1** Mapping of Illumina-only assembly bin205 (blue) to Illumina-PacBio hybrid assembly bin20 (red). Corresponding areas are connected in green.
