# Supplementary material for: Metagenomic binning of a marine sponge microbiome reveals unity in defense but metabolic specialization
Source: ISME J. 2017 Jul 11;11(11):2465–78. doi: 10.1038/ismej.2017.101 (PMC5649159; doi:10.1038/ismej.2017.101)
Supplement: Supplementary Figure S2 [file ismej2017101x2.docx]

**Figure S2** iTag analysis of the six DNA extracts for Illumina sequencing differing in cell lysis. Abbreviations: BB – beat beating, PK – proteinase K digestion, FT – freeze-thaw cycles.
