# Supplementary material for: Metagenomic binning of a marine sponge microbiome reveals unity in defense but metabolic specialization
Source: ISME J. 2017 Jul 11;11(11):2465–78. doi: 10.1038/ismej.2017.101 (PMC5649159; doi:10.1038/ismej.2017.101)
Supplement: Supplementary Figure S3 [file ismej2017101x3.docx]

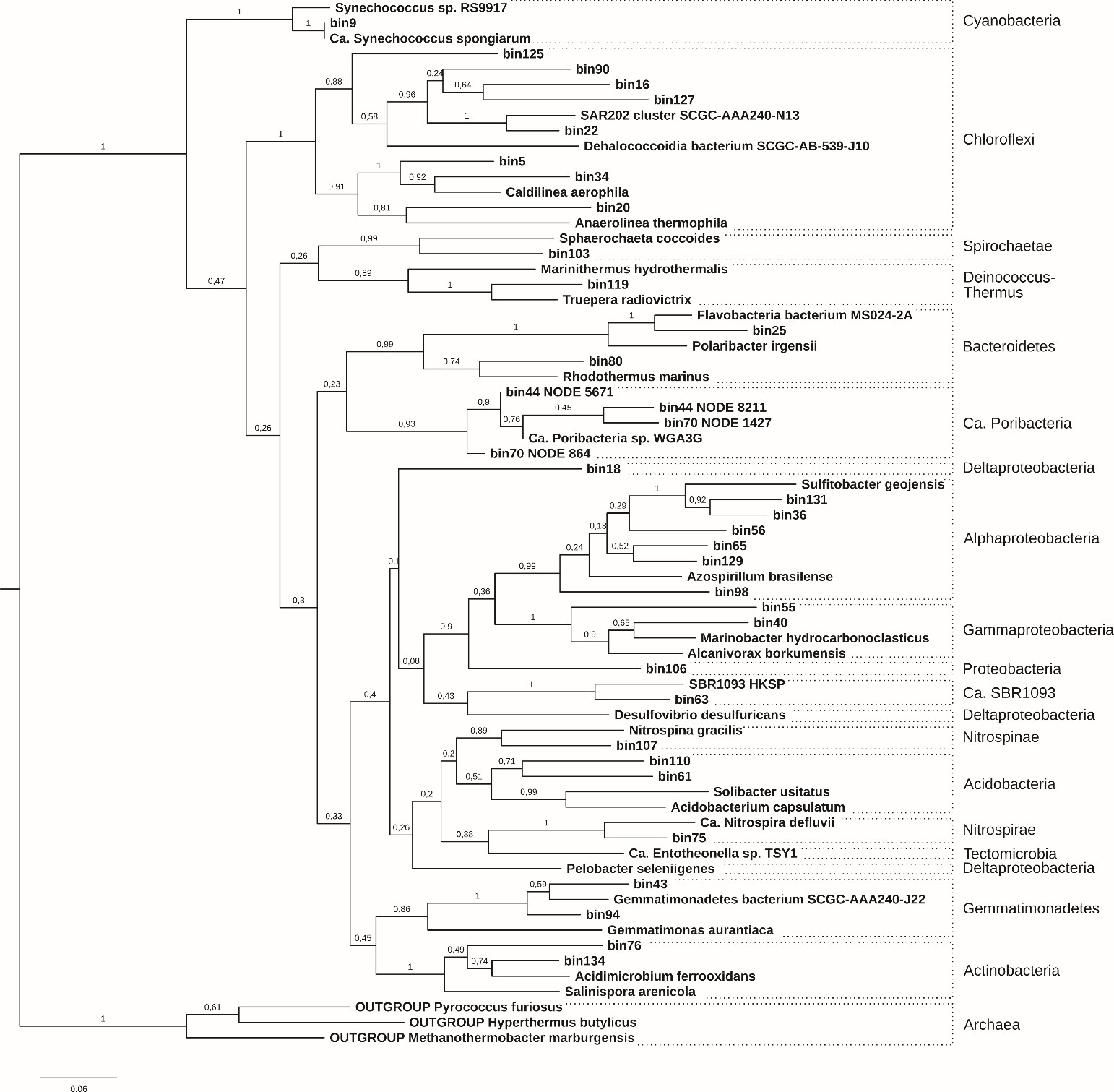


**Figure S3A** Neighbor Joining tree (GTR+G+I) with 100 bootstrap replications of 16S rRNA genes from hybrid assembly bins and their references. The following references were added to this tree only for better phylogenetic resolution: *Dehalococcoidia* bacterium SCGC-AB-539-J10 (ARPL01000017.1), “*Candidatus* Entotheonella” sp. TSY1 (KF926817.1), *Pyrococcus furiosus* (NR_074375.1), *Hyperthermus butylicus* (NR_102938.1), *Methanothermobacter marburgensis* (NR_028241.1).


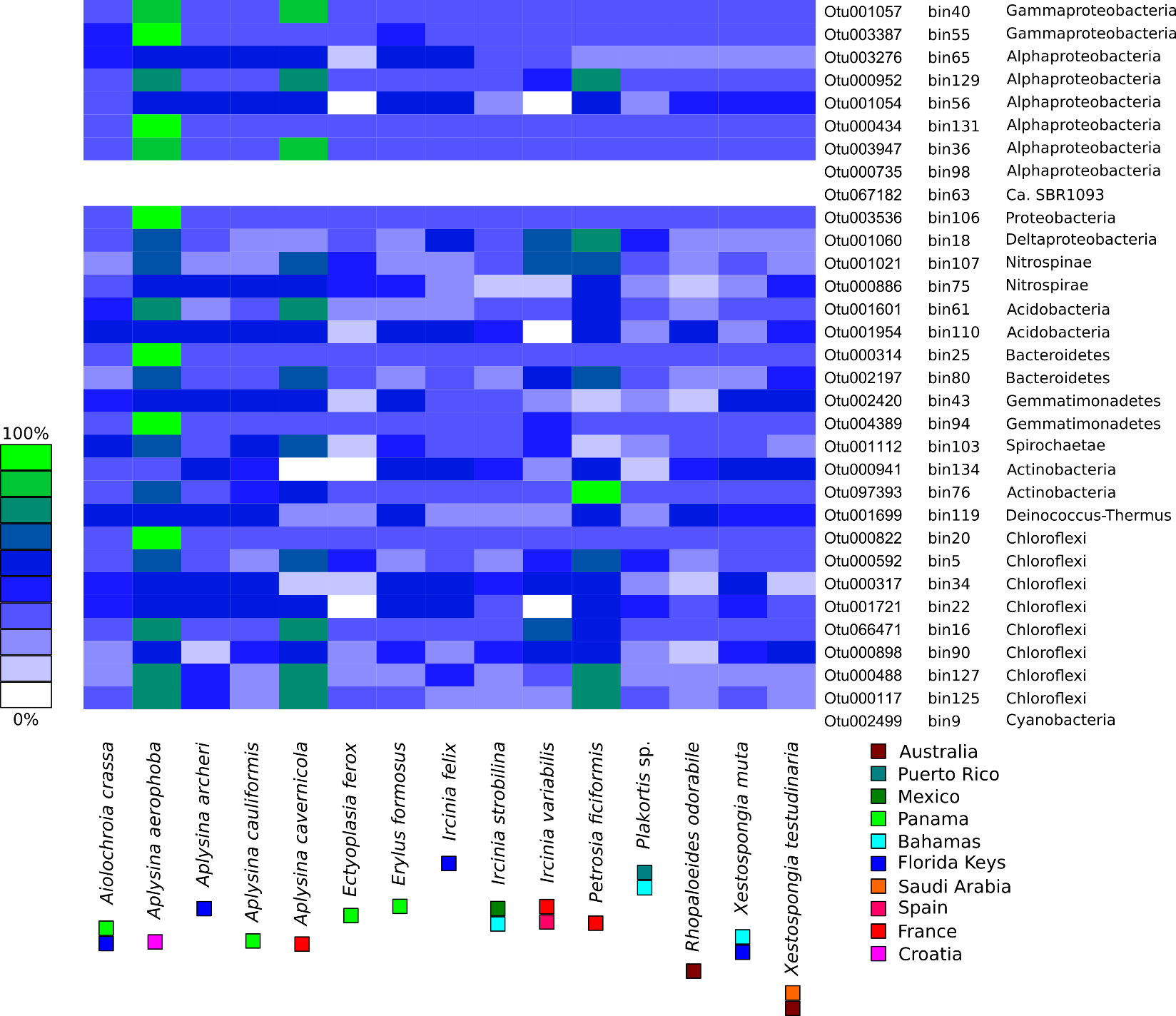


**Figure S3B** Distribution of representative OTUs in high microbial abundance (HMA) sponges in the amplicon dataset of Thomas et al. (2016). While the majority of OTUs is well represented in HMA sponges from various locations around the world, a concentration in Mediterranean species (from Spain, France, and Croatia) is apparent. According to the data of Thomas et al. (2016), OTU000735 seems to be specific for *Stylissa* sp. and especially low microbial abundance (LMA) sponge *Stylissa carteri*. Host species-specific bacterial communities as well as biogeographic patterns are a known feature in sponge microbiomes (Schmitt et al., 2012a). OTU067182 and OTU002499 were recovered mainly from sediments and also from seawater. Regarding cyanobacterial bin9 and OTU002499, it has to be noted that the short amplicon fragments of the 16S rRNA gene have been shown to be insufficient for phylogenetic placement of *Synechococcus* (Moitinho-Silva et al., 2014). Based on full-length 16S rRNA gene sequences, we were able to clearly identify bin9 as the sponge-specific “*Candidatus* Synechococcus spongiarum,” which is abundant and widespread in sponges (Erwin and Thacker, 2008).

**References**

Erwin PM, Thacker RW. (2008). Cryptic diversity of the symbiotic cyanobacterium *Synechococcus spongiarum* among sponge hosts. *Mol Ecol* **17**: 2937-2947. doi:10.1111/j.1365-294X.2008.03808.x.

Moitinho-Silva L, Bayer K, Cannistraci CV, Giles EC, Ryu T, Seridi L *et al*. (2014). Specificity and transcriptional activity of microbiota associated with low and high microbial abundance sponges from the Red Sea. *Mol Ecol* **23**: 1348-1363. doi:10.1111/mec.12365.

Schmitt S, Tsai P, Bell J, Fromont J, Ilan M, Lindquist N *et al*. (2012a). Assessing the complex sponge microbiota: Core, variable and species-specific bacterial communities in marine sponges. *ISME J* **6**: 564-576. doi:10.1038/ismej.2011.116.

Thomas T, Moitinho-Silva L, Lurgi M, Björk JR, Easson C, Astudillo-García C *et al*. (2016). Diversity, structure and convergent evolution of the global sponge microbiome. *Nat Commun* **7**: 11870. doi:10.1038/ncomms11870.
