# Supplementary material for: Metagenomic binning of a marine sponge microbiome reveals unity in defense but metabolic specialization
Source: ISME J. 2017 Jul 11;11(11):2465–78. doi: 10.1038/ismej.2017.101 (PMC5649159; doi:10.1038/ismej.2017.101)
Supplement: Supplementary Figure S4 [file ismej2017101x4.docx]

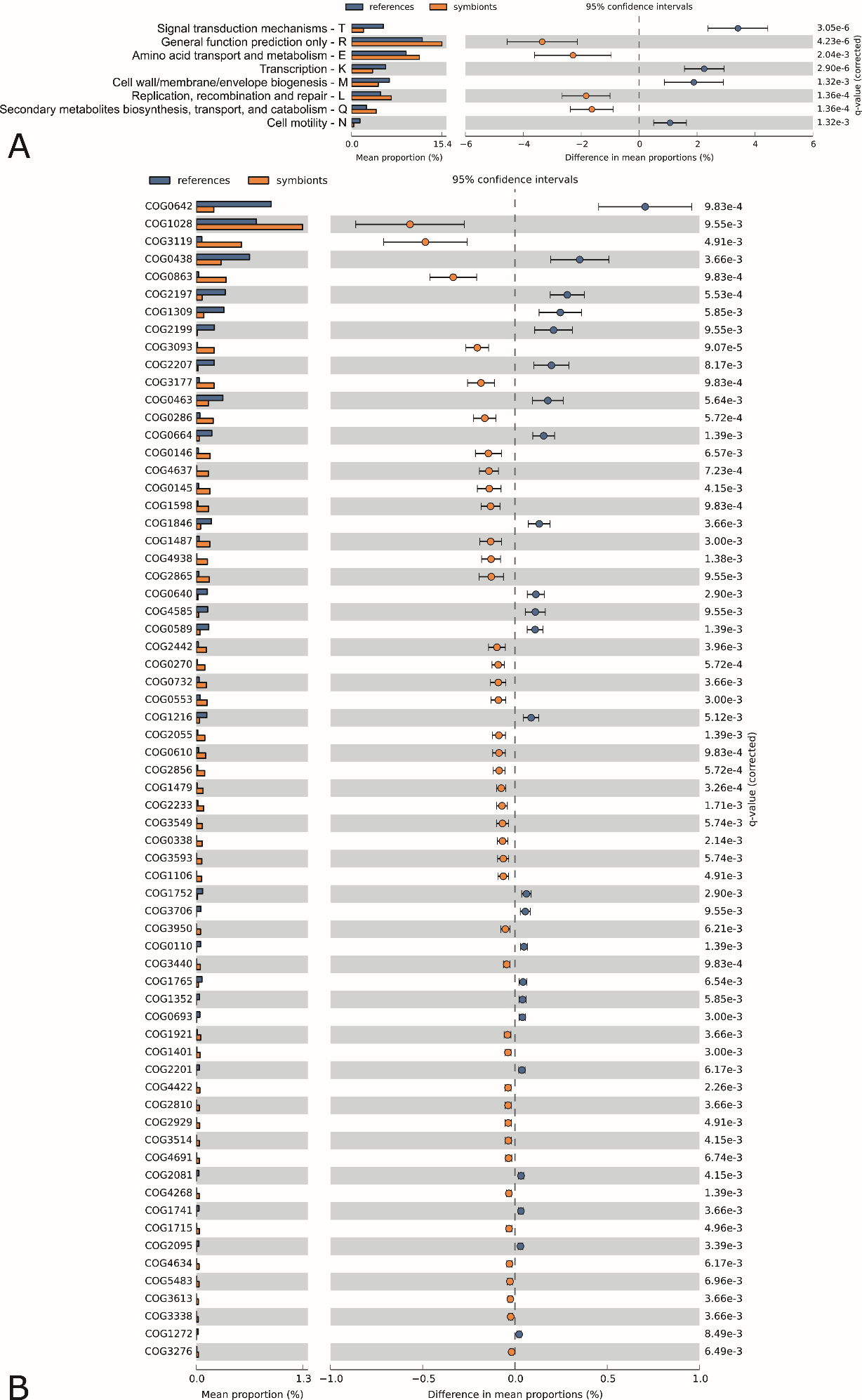


**Figure S4** Welch’s t-test with Storey FDR at a *q*-value cutoff of 0.01 and a confidence interval of 95% based on A) COG classes and B) COGs.
