# Supplementary material for: Metagenomic binning of a marine sponge microbiome reveals unity in defense but metabolic specialization
Source: ISME J. 2017 Jul 11;11(11):2465–78. doi: 10.1038/ismej.2017.101 (PMC5649159; doi:10.1038/ismej.2017.101)
Supplement: Supplementary Figure S5 [file ismej2017101x5.docx]

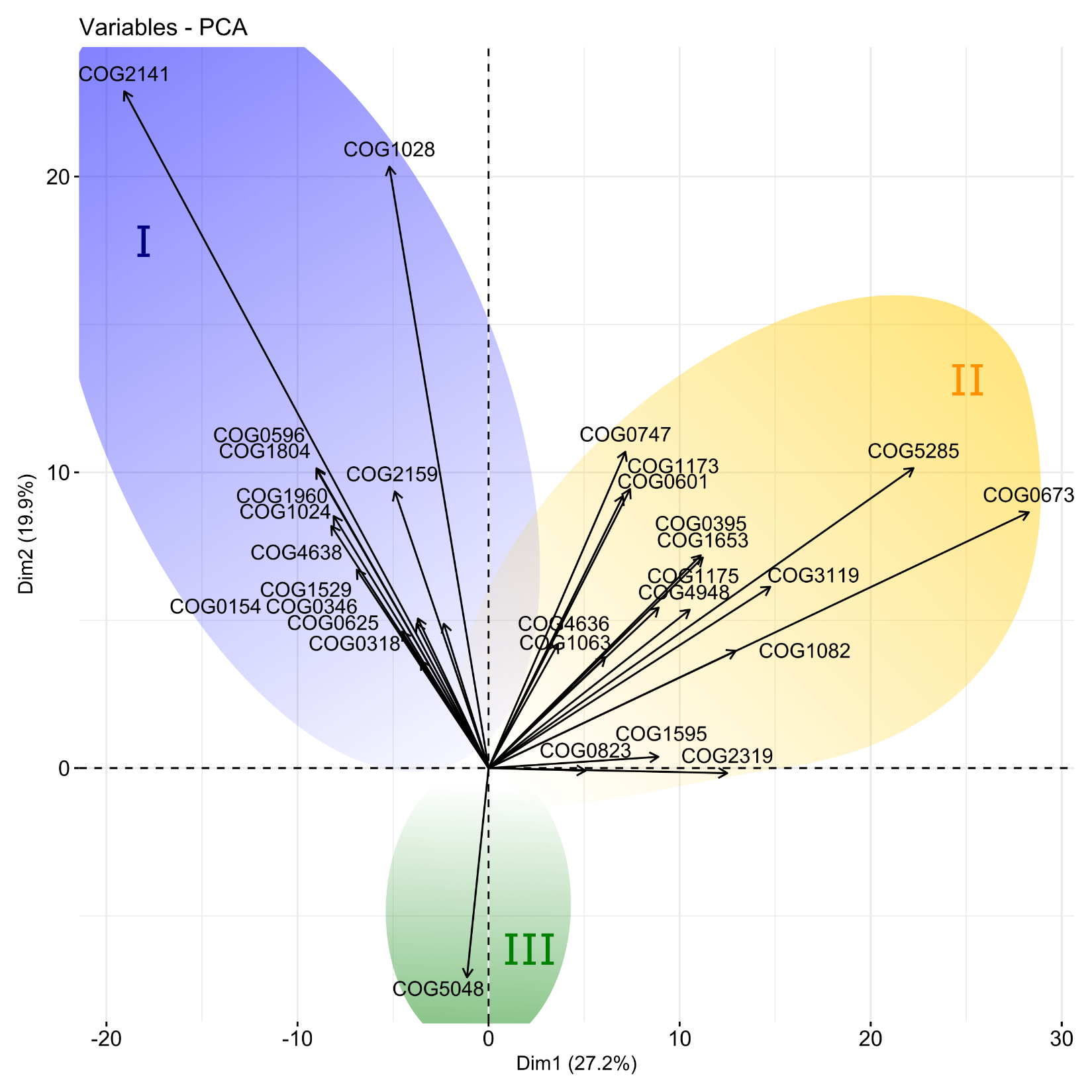


**Figure S5** The 30 COGs with the strongest influence on the PCA grouping of the sponge-symbiont genomes.
