# Supplementary figures and images for: Metagenomic binning of a marine sponge microbiome reveals unity in defense but metabolic specialization
Source: ISME J. 2017 Jul 11;11(11):2465–78. doi: 10.1038/ismej.2017.101 (PMC5649159; doi:10.1038/ismej.2017.101)

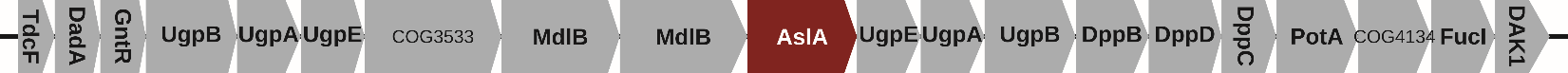


**Figure S6** Typical gene cluster around the arylsulfatase A (AslA, shown in red).

Supplement: Supplementary Figure S6 [file ismej2017101x6.docx]
