# Supplementary material for: Metagenomic binning of a marine sponge microbiome reveals unity in defense but metabolic specialization
Source: ISME J. 2017 Jul 11;11(11):2465–78. doi: 10.1038/ismej.2017.101 (PMC5649159; doi:10.1038/ismej.2017.101)
Supplement: Supplementary Table S1 [file ismej2017101x7.pdf]

Supplementary Table 1 Reference genomes for comparison with binned genomes of the PacBio-Illumina hybrid assembly.

Accession	Phylogeny	Source	% est. com.	Contig no.	Length (Mb)	% GC	N50	CDS no.	COG no.	% in COGs
NC_012483.1	Acidobacteria;Acidobacteria;Acidobacteriales;Acidobacteriaceae (Subgroup 1);Acidobacterium;*Acidobacterium capsulatum* ATCC51196	acidic mine drainage	94.59	1	4.1	60.50	4 127 356	3 343	2 290	68.50
NC_008536.1	Acidobacteria;Acidobacteria;Subgroup 3;unknown;Candidatus Solibacter;*Candidatus* Solibacter usitatus Ellin6076	pasture (soil)	93.69	1	10.0	61.90	9 965 640	8 102	4 755	58.69
CP001631	Actinobacteria;Acidimicrobiia;Acidimicrobiales;Acidimicrobiaceae;Acidimicrobium;*Acidimicrobium ferrooxidans* DSM10331	hot spring runoff	86.49	1	2.2	68.29	2 158 157	2 029	1 419	69.94
NC_009953.1	Actinobacteria;Actinobacteria;Micromonosporales;Micromonosporaceae;Salinispora;*Salinispora arenicola* CNS-205	tropical marine sediment	96.40	1	5.8	69.52	5 786 361	4 977	3 074	61.76
NC_013501.1 - NC_013502.1	Bacteroidetes;Cytophagia;Order II;Rhodothermaceae;Rhodothermus;*Rhodothermus marinus* DSM4252	shallow marine hot spring	95.50	2	3.4	64.30	3 261 604	2 918	2 053	70.36
NZ_ABVV01000000.1	Bacteroidetes;Flavobacteriia;Flavobacteriales;Flavobacteriaceae;NS5 marine group;*Flavobacteria bacterium* MS024-2A	coastal marine bacterium	88.29	17	1.9	35.71	371 257	1 762	1 221	69.30
NZ_CH724148.1 NZ_AAOG01000007.1	Bacteroidetes;Flavobacteriia;Flavobacteriales;Flavobacteriaceae;Polaribacter;*Polaribacter irgensii* 23-P	Antarctic surface water	92.79	2	2.8	34.52	2 753 184	2 423	1 583	65.33
NC_014960.1	Chloroflexi;Anaerolineae;Anaerolineales;Anaerolineaceae;Anaerolinea;*Anaerolinea thermophila* UNI-1	thermophilic granular sludge	93.69	1	3.5	53.85	3 532 378	3 161	2 227	70.45
NC_017079.1	Chloroflexi;Caldilineae;Caldilineales;Caldilineaceae;Caldilinea;*Caldilinea aerophila* DSM14535	NA	95.50	1	5.1	58.77	5 144 873	4 127	3 059	74.12
AQTZ01000000.1	Chloroflexi;SAR202 clade;SAR202 cluster bacterium SCGC-AAA240-N13	HOT station ALOHA	25.23	211	1.5	55.02	12 400	1 567	933	59.54
JYFQ01000000.1	Cyanobacteria;Cyanobacteria;SubsectionI;FamilyI;Candidatus Synechococcus spongiarum 15L	*Aplysina aerophoba*	89.19	229	2.2	59.16	14 814	2 276	1 290	56.68
CH724158.1	Cyanobacteria;Cyanobacteria;SubsectionI;FamilyI;Synechococcus;Synechococcus sp. RS9917	Eliat, Red Sea, 10m depth	94.60	1	2.6	64.46	2 584 918	2 738	1 630	59.53
NC_014221.1	Deinococcus-Thermus;Deinococci;Deinococcales;Trueperaceae;Truepera;*Truepera radiovictrix* DSM17093	hot spring runoff	92.79	1	3.3	68.14	3 260 398	2 995	2 174	72.59
NC_015387.1	Deinococcus-Thermus;Deinococci;Thermales;Thermaceae;Marinithermus;*Marinithermus hydrothermalis* DSM14884	deep-sea hydrothermal vent chimney	93.69	1	2.3	68.08	2 269 167	2 249	1 695	75.37
AXVX01000000.1	Gemmatimonadetes;Gemmatimonadetes;BD2-11 terrestrial group;Gemmatimonadetes bacterium SCGC-AAA240-J22	dark ocean below 200m	49.55	58	0.6	51.60	17 295	623	477	76.57
NC_012489.1	Gemmatimonadetes;Gemmatimonadetes;Gemmatimonadales;Gemmatimonadaceae;Gemmatimonas;*Gemmatimonas aurantiaca* T-27	anaerobic-aerobic sequential batch reactor	94.59	1	4.6	64.27	4 636 964	3 968	2 593	65.35
NC_014355.1	Nitrospirae;Nitrospira;Nitrospirales;Nitrospiraceae;Nitrospira;Candidatus Nitrospira defluvii	municipal wastewater treatment plant	93.69	1	4.3	59.03	4 317 083	4 119	2 514	61.03
ASZN01000000.1	Candidatus Poribacteria;Poribacteria genera incertae sedis;Candidatus Poribacteria WGA3G	*Aplysina aerophoba*	82.88	283	5.4	47.79	51 110	4 716	2 693	57.10
NZ_JASE01000000.1	Proteobacteria;Alphaproteobacteria;Rhodobacterales;Rhodobacteraceae;Sulfitobacter;*Sulfitobacter geojensis*	coastal seawater	94.59	5	4.2	57.84	3 790 153	3 990	3 101	77.72
NC_016617.1	Proteobacteria;Alphaproteobacteria;Rhodospirillales;Rhodospirillaceae;Azospirillum;*Azospirillum brasilense* Sp245	rhizosphere and plant tissues	91.89	7	7.5	68.49	1 766 028	6 985	4 852	69.46
NC_016803.1	Proteobacteria;Deltaproteobacteria;Desulfovibrionales;Desulfovibrionaceae;Desulfovibrio;*Desulfovibrio desulfuricans* ND132	found in soil, freshwater, saltwater, intestinal tract of animals	93.69	1	3.9	65.21	3 858 580	3 515	2 578	73.34
NZ_JOMG01000000.1	Proteobacteria;Deltaproteobacteria;Desulfuromonadales;Desulfuromonadaceae;Pelobacter;*Pelobacter seleniigenes* DSM18267	wetlands, freshwater	91.89	6	5.1	54.19	3 076 292	4 448	3 354	75.40
NC_008738.1 – NC_008740.1	Proteobacteria;Gammaproteobacteria;Alteromonadales;Alteromonadaceae;Marinobacter;*Marinobacter hydrocarbonoclasticus* VT8	hydrocarbon polluted marine environments, survives in open seawater	95.50	3	4.8	56.93	4 326 849	4 453	3 161	70.99
NC_008260.1	Proteobacteria;Gammaproteobacteria;Oceanospirillales;Alcanivoracaceae;Alcanivorax;*Alcanivorax borkumensis* SK2	seawater sediment, North Sea	94.59	1	3.1	54.73	3 120 143	2 793	2 239	80.16
NC_015436.1	Spirochaetae;Spirochaetes;Spirochaetales;Spirochaetaceae;Sphaerochaeta;*Sphaerochaeta coccoides* DSM17374	termite hindgut	89.19	1	2.2	50.56	2 227 296	1 913	1 425	74.49
NZ_HG422173.1 - NZ_HG422176.1	Nitrospinae/Tectomicrobia group;Nitrospinae;Nitrospinia;Nitrospinales;Nitrospinaceae;Nitrospina;*Nitrospina gracilis* 3/211	seawater	92.79	4	3.1	56.21	3 072 611	2 934	1 940	66.12
mgm4539207.3	SBR1093;clade I;SBR1093 HKSP	industrial wastewater treatment plant	89.19	184	2.7	56.49	26 372	2 585	1 802	69.71

Abbreviations: est. - estimated; com. - completeness; no. - number
