# Supplementary material for: Metagenomic binning of a marine sponge microbiome reveals unity in defense but metabolic specialization
Source: ISME J. 2017 Jul 11;11(11):2465–78. doi: 10.1038/ismej.2017.101 (PMC5649159; doi:10.1038/ismej.2017.101)
Supplement: Supplementary Table S3 [file ismej2017101x9.pdf]

Supplementary Table 3A Best BLAST hits for the 16S rRNA genes of the PacBio-Illumina hybrid assembly bins.

Genome	Query coverage	Identity	BLAST hit accession	description	Sponge-derived	Sponge species
**bin131**	94%	94%	JX206636.1	Uncultured bacterium clone TO10-919_C31	yes	*Ircinia oros*
**bin56**	95%	99%	JX206526.1	Uncultured bacterium clone AF10-915_C9	yes	*Ircinia fasciculata*
**bin52**	no 16S rRNA gene available					
**bin65**	95%	99%	JX206637.1	Uncultured bacterium clone TO10-919_C32	yes	*Ircinia oros*
**bin36**	94%	97%	EF076136.1	Uncultured alpha proteobacterium clone AD015	yes	*Agelas dilatata*
**bin98**	86%	92%	EU350888.1	Uncultured alpha proteobacterium clone HAL-T-35	yes	*Haliclona simulans*
**bin129**	98%	99%	HE817825.1	Uncultured bacterium partial 16S rRNa gene, clone B293/GW947	yes	*Vaceletia crypta*
**bin95**	no 16S rRNA gene available					
**bin107**	94%	99%	JX206591.1	Uncultured bacterium clone TO10-97_C15	yes	*Ircinia oros*
**bin63**	97%	99%	JN002375.1	Uncultured microorganism clone WGA_alt_1-4E-16S(clone3-3)	yes	*Aplysina aerophoba*
**bin18**	97%	99%	JX206694.1	Uncultured bacterium clone TV10-97_C2	yes	*Ircinia variabilis*
**bin40**	99%	92%	CP013251.1	*Endozoicomonas montiporae* CL-33	no (coral)	-
	97%	92%	KC669143.1	Uncultured bacterium clone 15E04	no (coral)	-
	97%	92%	AB205011.1	*Spongiobacter nickelotolerans* gene for 16S rRNA	yes	-
**bin55**	90%	93%	KF286003.1	Uncultured gamma proteobacterium clone CtgComparison_34	yes	*Aplysina cauliformis*
**bin106**	83%	98%	AM259914.1	Uncultured proteobacterium partial 16S rRNA gene, clone CN28	yes	*Chondrilla nucula*
**bin75**	98%	99%	JQ359623.1	Uncultured bacterium clone bac37	yes	*Xestospongia testudinaria*
**bin110**	95%	99%	JX206593.1	Uncultured bacterium clone TO10-97_C17	yes	*Ircinia oros*
**bin61**	97%	99%	FJ269286.1	Uncultured Acidobacteria bacterium clone XA2H05F	yes	*Xestospongia testudinaria*
**bin70 (NODE_864)**	100%	96%	AY713479.1	Uncultured Poribacteria bacterium 64K2	yes	-
**bin70 (NODE_1427)**	97%	96%	AY713479.1	Uncultured Poribacteria bacterium 64K2	yes	-
**bin44 (NODE_5671)**	97%	99%	AY713479.1	Uncultured Poribacteria bacterium 64K2	yes	-
**bin44 (NODE_8211)**	99%	97%	AY713479.1	Uncultured Poribacteria bacterium 64K2	yes	-
**bin80**	97%	99%	JX206706.1	Uncultured bacterium clone TV10-97_C25	yes	*Ircinia variabilis*
**bin25**	96%	98%	JN655253.1	Uncultured bacterium clone AF10-3-9_C14	yes	*Ircinia fasciculata*
**bin43**	95%	98%	HQ270243.1	Uncultured bacterium clone XA2F08F	yes	*Xestospongia testudinaria*
**bin94**	94%	98%	JX280155.1	Uncultured bacterium clone BA01-C14-seq	yes	*Ircinia felix* tan morph
**bin103**	95%	98%	JQ612254.1	Uncultured bacterium clone GBc085	yes	*Geodia barretti*
**bin76**	98%	99%	KC669080.1	Uncultured bacterium clone 14H01	no (coral)	-
	98%	98%	FJ229928.1	Uncultured actinobacterium clone XA3F02F	yes	*Xestospongia testudinaria*
**bin134**	95%	99%	JX206600.1	Uncultured bacterium clone TO10-97_C25	yes	*Ircinia oros*
**bin119**	88%	99%	HQ270284.1	Uncultured *Truepera* sp. Clone XE1D04	yes	*Xestospongia muta*
**bin9**	99%	99%	KJ174471.1	*Candidatus* Synechococcus spongiarum SH4	yes	*Carteriospongia foliascens*
**bin5**	99%	99%	FJ560485.1	Uncultured Chloroflexi bacterium li19	yes	*Aplysina aerophoba*
**bin34**	97%	99%	JX206705.1	Uncultured bacterium clone TV10-97_C23	yes	*Ircinia variabilis*
**bin22**	95%	98%	EF076083.1	Uncultured Chloroflexi bacterium clone PK016	yes	*Plakortis* sp.
**bin127**	95%	98%	JQ612181.1	Uncultured bacterium clone GBc144	yes	*Geodia barretti*
**bin90**	97%	98%	HE985083.1	Uncultured bacterium partial 16S rRNA gene, clone A48/GW950	yes	*Astrosclera willeyana*
**bin16**	95%	99%	JX206718.1	Uncultured bacterium clone TV10-912_C6	yes	*Ircinia variabilis*
**bin125**	95%	98%	FJ481340.1	Uncultured *Chloroflexus* sp. Clone XB3G04F	yes	*Xestospongia muta*
**bin20**	87%	99%	AJ347043.1	Uncultured bacterium 16S rRNa gene, clone TK35	yes	*Aplysina aerophoba*
**bin87**	no 16S rRNA gene available					

Supplementary Table 3B Closest reference genomes to bins based on 16S rRNA genes.

Genome	Closest reference genome	Identity	Alignment length
**bin103**	*Sphaerochaeta coccoides* DSM17374	84.23	1547
**bin106**	*Alcanivorax borkumensis* SK2	81.77	1563
**bin107**	*Nitrospina gracilis* NZ_HG422173.1.2	85.31	1572
**bin110**	*Acidobacterium capsulatum* ATCC51196	84.99	1079
**bin119**	*Truepera radiovictrix* DSM17093	88.71	1523
**bin125**	*Caldilinea aerophila* NC_017079.1	82.99	1523
**bin127**	SAR202 cluster SCGC-AAA240-N13	82.79	1528
**bin129**	*Azospirillum brasilense* Sp245	87.94	1236
**bin131**	*Sulfitobacter geojensis*	87.55	1494
**bin134**	*Acidimicrobium ferrooxidans* DSM10331	89.5	1515
**bin16**	SAR202 cluster SCGC-AAA240-N13	83.57	1534
**bin18**	*Desulfovibrio desulfuricans* ND132	80.47	901
**bin20**	*Anaerolinea thermophila* UNI-1	81.45	1531
**bin22**	SAR202 cluster SCGC-AAA240-N13	91.69	1517
**bin25**	Flavobacteria bacterium MS024-2A	91.17	1529
**bin34**	*Caldilinea aerophila* NC_017079.1	87.6	1508
**bin36**	*Sulfitobacter geojensis*	89.64	1486
**bin40**	*Marinobacter hydrocarbonoclasticus* VT8	88.76	1539
**bin43**	Gemmatimonadetes bact. SCGC-AAA240-J22	92.2	1565
**bin44 NODE 5671**	Ca. Poribacteria sp. WGA3G KC713965.1	98.49	199
**bin44 NODE 8211**	Dehalococcoidia bact. SCGC-AB-539-J10	84.39	173
**bin5**	*Caldilinea aerophila* NC_017079.1	87.39	1522
**bin55**	*Marinobacter hydrocarbonoclasticus* VT8	85.35	1386
**bin56**	*Azospirillum brasilense* Sp245	87.08	1501
**bin61**	*Nitrospina gracilis* NZ_HG422173.1.2	82.24	1571
**bin63**	SBR1093 genome 210566.2	88.39	1567
**bin65**	*Azospirillum brasilense* Sp245	88.84	1496
**bin70 NODE 1427**	Dehalococcoidia bact. SCGC-AB-539-J10	86.54	156
**bin70 NODE 864**	Ca. Poribacteria sp. WGA3G KC713965.1	95.48	199
**bin75**	*Ca.* Nitrospira defluvii	89.21	1548
**bin76**	*Acidimicrobium ferrooxidans* DSM10331	86.29	1087
**bin80**	*Rhodothermus marinus* DSM4252	86.96	1541
**bin9**	*Ca.* Synechococcus spongiarum 15L	100	1491
**bin9**	*Synechococcus* sp. RS9917	95.23	1489
**bin90**	SAR202 cluster SCGC-AAA240-N13	84.06	1524
**bin94**	Gemmatimonadetes bact. SCGC-AAA240-J22	92.02	1566
**bin98**	*Azospirillum brasilense* Sp245	83.48	1029
