# Supplementary material for: Metagenomic binning of a marine sponge microbiome reveals unity in defense but metabolic specialization
Source: ISME J. 2017 Jul 11;11(11):2465–78. doi: 10.1038/ismej.2017.101 (PMC5649159; doi:10.1038/ismej.2017.101)
Supplement: Supplementary Table S5 [file ismej2017101x11.pdf]

Supplementary Table 5 Most common COGs within 10 genes before and after arylsulfatase genes in all clusters of more than 10 genes.

COG	description	SUM	in X of 282 clusters	in % of clusters
COG3119	AslA, Arylsulfatase A and related enzymes	397	282	100.00%
COG0673	MviM, Predicted dehydrogenases and related proteins	136	98	34.75%
COG1028	FabG, Dehydrogenases with different specificities (related to short-chain alcohol dehydrogenases)	86	72	25.53%
COG5285	COG5285, Protein involved in biosynthesis of mitomycin antibiotics/polyketide fumonisin	107	69	24.47%
COG1653	UgpB, ABC-type sugar transport system, periplasmic component	68	51	18.09%
COG1173	DppC, ABC-type dipeptide/oligopeptide/nickel transport systems, permease components	64	48	17.02%
COG0747	DdpA, ABC-type dipeptide transport system, periplasmic component	69	48	17.02%
COG0601	DppB, ABC-type dipeptide/oligopeptide/nickel transport systems, permease components	63	47	16.67%
COG4948	COG4948, L-alanine-DL-glutamate epimerase and related enzymes of enolase superfamily	67	46	16.31%
COG0395	UgpE, ABC-type sugar transport system, permease component	64	46	16.31%
COG1082	IolE, Sugar phosphate isomerases/epimerases	54	46	16.31%
COG1175	UgpA, ABC-type sugar transport systems, permease components	53	39	13.83%
